# Strengthening Effect of Extruded Mg-8Sn-2Zn-2Al Alloy: Influence of Micro and Nano-Size Mg_2_Sn Precipitates

**DOI:** 10.3390/ma10070822

**Published:** 2017-07-18

**Authors:** Weili Cheng, Yang Bai, Lifei Wang, Hongxia Wang, Liping Bian, Hui Yu

**Affiliations:** 1 Shanxi Key Laboratory of Advanced Magnesium-Based Materials, Taiyuan University of Technology, Taiyuan 030024, China; Lifeiwang6@gmail.com (L.W.); wanghxia1217@163.com (H.W.); bianliping_724@126.com (L.B.); 2 Key Laboratory of Interface Science and Engineering in Advanced Materials, Ministry of Education, Taiyuan University of Technology, Taiyuan 030024, China; 3 School of Materials Science and Engineering, Taiyuan University of Technology, Taiyuan 030024, China; baiyang0087@link.tyut.edu.cn; 4 School of Materials Science and Engineering, Hebei University of Technology, Tianjin 300132, China

**Keywords:** Mg-Sn alloy, extrusion, microstructure, precipitation, strengthening

## Abstract

In this study, Mg-8Sn-2Zn-2Al (TZA822) alloys with varying Mg_2_Sn contents prior to extrusion were obtained by different pre-treatments (without and with T4), and the strengthening response related to micro and nano-size Mg_2_Sn precipitates in the extruded TZA822 alloys was reported. The results showed that the morphology of nano-size Mg_2_Sn precipitates exhibits a significant change in basal plane from rod-like to spherical, owing to the decrement in the fraction of micro-size particles before extrusion. Meanwhile, the spherical Mg_2_Sn precipitates provided a much stronger strengthening effect than did the rod-like ones, which was ascribed to uniform dispersion and refinement of spherical precipitates to effectively hinder basal dislocation slip. As a consequence, the extruded TZA822 alloy with T4 showed a higher tensile yield strength (TYS) of 245 MPa, ultimate tensile strength (UTS) of 320 MPa and elongation (EL) of 26.5%, as well as a lower degree of yield asymmetry than their counterpart without T4. Detailed reasons for the strengthening effect were given and analyzed.

## 1. Introduction

Recently, due to the variety of inherent merits such as creep resistance, superplastic behavior at elevated temperatures and specifically good extrudability, Mg-Sn based alloys have attracted great interests, particularly in the extruding industry [1,2]. This is mainly attributed to the high solidus temperature of Mg_2_Sn intermetallic, which could prevent the occurrence of hot shortness when comparing with Mg-Zn and Mg-Al based alloys [3]. However, the application of binary Mg-Sn alloy is still very limited due to the relatively low absolute strength. Micro-alloying is usually regarded as an effective method for strengthening Mg alloys. Various alloying elements, such as Zn [4,5], Na and Li [6], Ca [7] and Ag [8] are added to Mg-Sn alloys to improve their strength by grain refinement. Among the above-mentioned elements, Zn has been found not only to refine the grain size but also to promote the formation of Mg_2_Sn precipitates in the Mg matrix [9]. Furthermore, many studies have reported that extruded Mg-Sn-Zn-Al (TZA) alloys exhibit a greater strength than that of commercial Mg-Zn and Mg-Al based alloys [10,11].

Besides alloying, precipitation strengthening is also a promising way to achieve both improved strength and ductility. It is known that the precipitation-hardening of Mg alloys is influenced not only by the particles’ volume fraction, but also by the morphology and orientation of particles. For example, a previous study reported that tensile and compressive strengths enhancements of Mg-Sn binary alloys were mainly attributable to the increase in volume fraction of Mg_2_Sn precipitates as the Sn content increased [1]. In randomly textured materials, Mg-Al-Zn (AZ) alloys with basal plate precipitates generally give poor strengthening when compared to the prismatic plates that form in Mg-Y-RE (rare-earth) alloys [12]. This difference indicated that the increment of the critical resolved shear stress (CRSS) for basal slip generated by prismatic plates is larger than that generated by basal plates [13]. Sasaki et al. developed a Mg-Sn-Zn-Al (TZA) alloy that shows both high strength and good ductility compare with other previously reported wrought Mg alloys [11]. The significant strengthening by T6 treatment was ascribed to uniform distribution of precipitates and their increase in number density. However, there was a lack of quantitative discussion of strengthening response due to fine Mg_2_Sn particles in the extruded alloys. In addition, Park et al. found that the formation of twins in TZA811 alloy by cold forging prior to extrusion is propitious to promoting dynamic recrystallization (DRX) nucleation and increment of Mg_2_Sn precipitates, thereby increasing DRX fraction and strength [14]. This indicated that the state of the initial billet plays an important role in the strengthening effect of extruded Mg alloys. 

Even though lots of work has been done, detailed information about the dependence of DRX behavior and precipitation strengthening on the volume fraction of the remaining particles in the Mg-Sn based alloys is relatively limited. Therefore, the present work aims to discuss the role of the Mg_2_Sn precipitates in the Mg-Sn-Zn-Al alloy system in order to pave the way for the development of high-strength RE-free Mg alloys, and reveal the micro and nano-size Mg_2_Sn precipitates dependent strengthening effect in extruded Mg-8Sn-2Zn-2Al alloy.

## 2. Experimental Procedure

The ingots with nominal composition Mg-8Sn-2Zn-2Al (wt %) (TZA822) were prepared by melting commercially pure Mg (99.9 wt %), Sn (99.99 wt %), Zn (99.99 wt %) and Al (99.99 wt %) in an electrical resistance furnace with the protection of a mixture of gaseous CO_2_ and SF_6_. After casting, solution treatment was conducted at 320 °C for 3 h and then 450 °C for 24 h, followed by water-quenching. The natural aging treatment was executed at 25 °C for 360 h. The samples (40 mm in diameter and 50 mm in length) were machined and extruded at an initial billet temperature of 300 °C, a ram speed of 0.1 mm·s^−1^ and an extrusion ratio of 16. The extruded TZA822 alloy with only cast treatment was marked as “extruded alloy without T4” and the alloy with solution treatment and natural aging treatment was designated as “extruded alloy with T4”. 

The microstructures of the specimens were examined by a Leica 2700 M optical microscope (OM, Lecia Microsystem GmbH, Wetzlar, Germany), a Mira 3XMU scanning electron microscope (SEM, TESCAN Ltd., Brno-Kohoutovice, Czech Republic) equipped with an energy dispersive spectrometer (EDS), and a JEM-2100F transmission electron microscope (TEM, JEOL Ltd., Tokyo, Japan). SEM images were taken in the secondary electron (SE) mode. The average grain size and the amounts of both DRXed grains and precipitates were calculated from the number and/or area fraction using three micrographs by the Image-Pro plus 6.0 software. (0002) and (10$\bar{1}$0) pole figures of the extruded samples were performed with a Y-2000 X-ray diffractometer (XRD, Cu-Ka, Dandong Ray Instrument Co., Ltd., Dandong, China). The tensile and compressive tests were carried out at room temperature using a DNS100 electric testing machine with an initial strain rate of 1 × 10^−3^ s^−1^ (SFMIT Ltd. Changzhou, China). The tensile specimens (dog-bone-shaped) were 18 mm gauge length, 4 mm gauge width and 2 mm gauge thickness, the compressive specimens (cylindrical) were 8 mm in diameter and 12 mm in height. These specimens were cut from the extruded rods along the extrusion direction (ED). The tensile and compressive tests for each specimen were repeated three times, and the average values of these measurements were used in this study.

## 3. Results and Discussion

### 3.1. The Microstructures Prior to Extrusion

**Figure 1:**
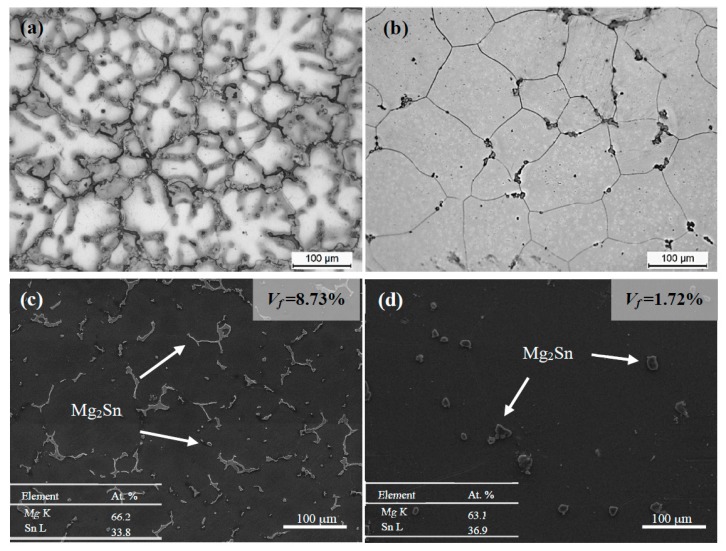
OM and SEM-SE images of TZA822 alloys prior to extrusion in different states: (**a**,**c**) without T4 and (**b**,**d**) with T4.

Figure 1 shows the microstructures of TZA822 alloys without and with T4 prior to extrusion. The alloy without T4 (Figure 1a) exhibited a typical dendritic microstructure, and the average secondary dendrite arm space was ~62 μm. After T4 (Figure 1b), the equiaxial grains with an average grain size of ~120 μm and a few undissolved particles distributed at the grain boundaries and grain interiors could be observed. According to the SEM and EDS results (Figure 1c,d), these undissolved particles could be identified as Mg_2_Sn phase. The volume fractions of these phases in both conditions were measured to be ~8.73% and ~1.72%, respectively. According to the Mg-Sn binary phase diagram, since the Mg_2_Sn phase has a melting point of 770 °C and the equilibrium solid solubility of Sn in Mg at 450 °C is approximately 7.28 wt %, few Mg_2_Sn phases should remain in the alloy after solution treatment. These differences in the volume fraction of Mg_2_Sn particles resulted in various extrusion morphologies, as well as related tensile properties, which will be discussed in the following sections.

### 3.2. The Microstructures after Extrusion

**Figure 2:**
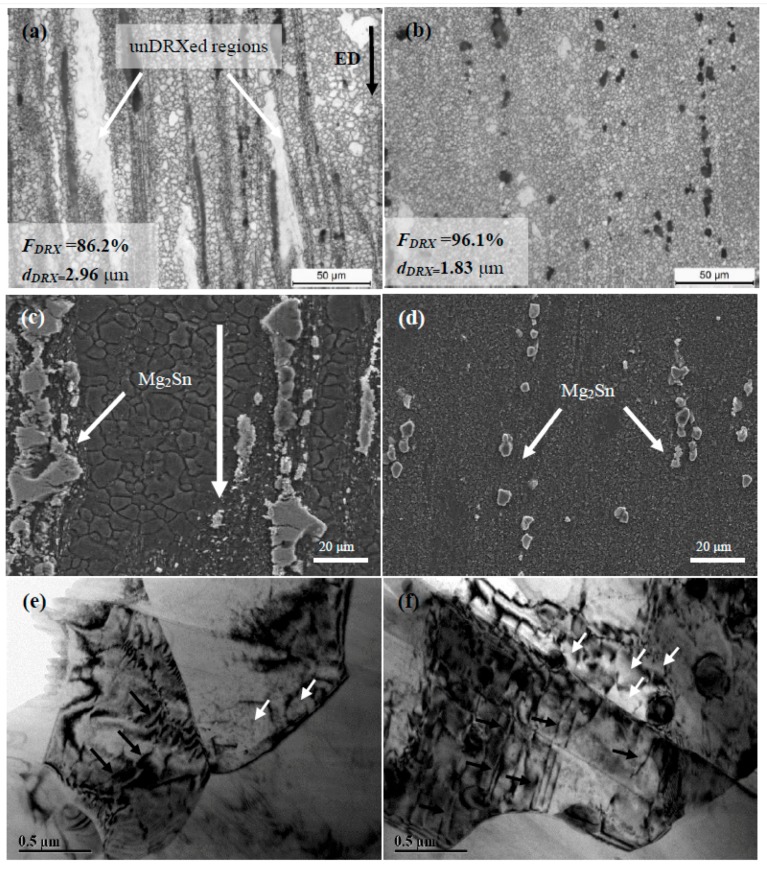
OM, SEM-SE and TEM images of extruded TZA822 alloys: (**a**,**c**,**e**) without T4 and (**b**,**d**,**f**) with T4, respectively.

Figure 2 shows the micrographs of extruded TZA822 alloys. It can be clearly seen that the extruded TZA822 alloys exhibit a typical bimodal structure, with fine and equiaxial recrystallized grains (DRXed grains) and coarse un-recrystallized grains elongated along the ED (unDRXed grains) (Figure 2a,b). Furthermore, the micro-size Mg_2_Sn particles presented in the samples without and with T4 were found to be aligned along the ED in the form of stringers after being broken into fragments during the extrusion process (arrows in Figure 2c,d). More detailed DRX behavior could be recognized by TEM analysis. As indicated (Figure 2e,f), the coarser grain consists of dislocation walls and sub-grains with low-angle grain boundaries (LAGBS), marked by black and white arrows, which might be attributable to dislocation climb or cross-slip [15]. Generally, discontinuous dynamic recrystallization (DDRX) and continuous dynamic recrystallization (CDRX) are the main DRX modes in the wrought Mg alloys. DDRX is mainly attributed to nucleation and nucleus growth by high-angle grain boundaries (HAGBS) migration. It results in a ‘necklace’ structure at the original grain boundaries. For CDRX, new grains originate in the transformation of sub-grains with LAGBS into HAGBS by continuously absorbing dislocations [15,16]. Moreover, previous reports indicate that, with deformation temperature higher than 300 °C and strain higher than 0.6, CDRX is the dominating DRX mechanism in wrought Mg alloys [16,17]. As mentioned above, the microstructure characteristics (sub-grains, dislocation, but with no ‘necklace’ structure) could be observed, and deformation temperature (300 °C) and strain (*ε* = 2.8 based on ε=lnER [18], where ER is the extrusion ratio) were suitable. Therefore, it can be concluded that CDRX was the dominating DRX mechanism. In addition, the measured area fraction and average sizes of DRXed grains (*F_DRX_* and *d_DRX_*) for both alloys were ~86.2%, ~2.96 μm and ~96.1%, ~1.83 μm, respectively. The detailed microstructural characteristics are also provided in Table 1. Thus, a more homogenous microstructure with finer dispersive Mg_2_Sn intermetallic could be obtained in the extruded alloy with T4 compared with the one without T4, indicating that the fraction of remaining precipitates plays an important role in DRX processing.

**Figure 3:**
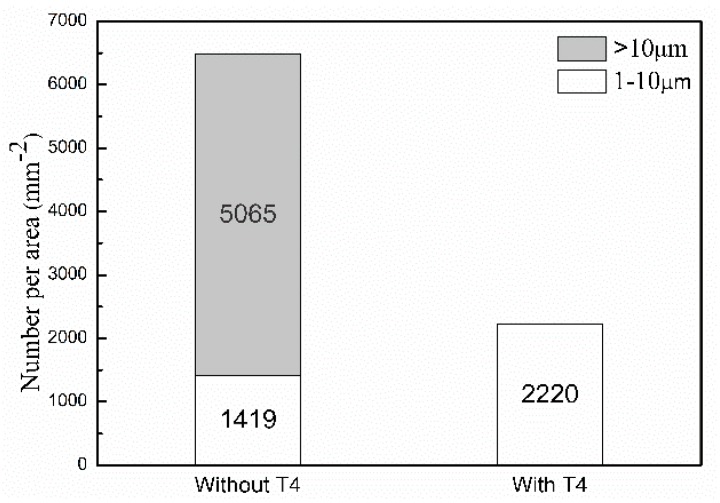
Particle-size distribution and number per area for the extruded TZA822 alloys.

The size distribution and number per area of Mg_2_Sn particles larger than 1 μm in the extruded TZA822 alloys are shown in Figure 3. As indicated, the number per area of particles ranging from 1 to 10 μm in size of the extruded alloy with T4 was larger than for their counterpart without T4. It is known that particles having 1~10 μm diameter can act as nucleation sites for DRX during hot deformation, because of the higher dislocation density and large orientation gradient induced at the deformed zones in the vicinity of the particles [19]. This phenomenon is known as particle stimulated nucleation (PSN), and has been widely observed in wrought Mg alloys [20,21]. For this reason, a larger amount of the 1~10 μm sized particles led to a higher fraction of DRXed grains in the extruded alloy with T4.

**Figure 4:**
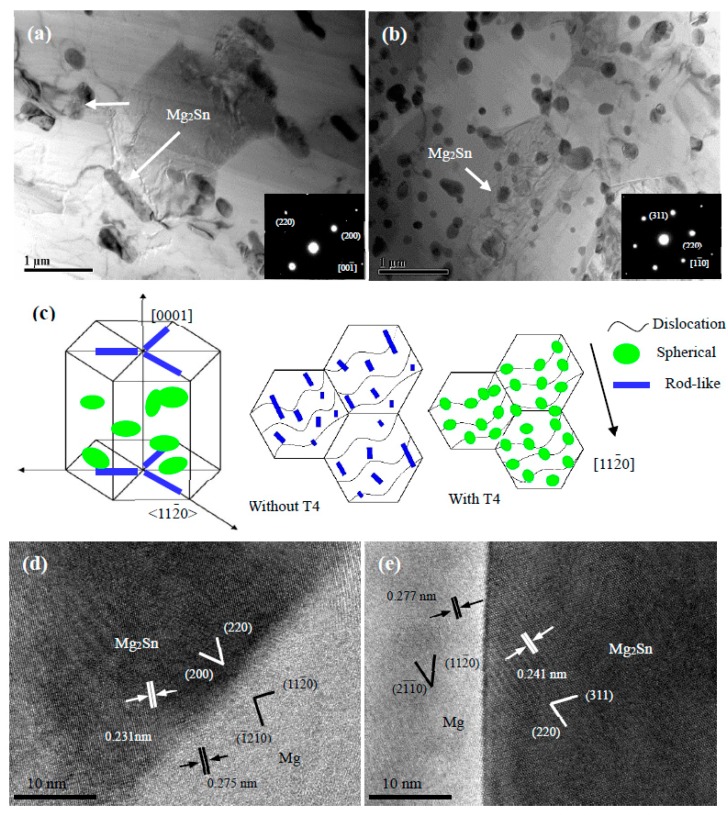
Bright field TEM images of DRXed region of extruded TZA822 alloys taken from the zone axis of [0001] (inset: SAED pattern) (**a**) without T4; (**b**) with T4; (**c**) The schematic illustration of the two different types of precipitates; (**d**,**e**) HR-TEM of rod-like and spherical precipitates.

In order to figure out the dynamic precipitation behavior of nano-size phase, TEM observation was performed. Figure 4a,b show the TEM images of DRXed regions in the extruded TZA822 alloys taken from the zone axis of [0001], which exhibited that numerous nano-size precipitates were distributed along the grain boundaries, as well as within the grain interiors. The volume fraction and average size of the precipitates for the both alloys were about 8.11%, 351.8 nm and 13.87%, 160.7 nm, respectively. Moreover, it was found that the decrease in amount of micro-size particles prior to extrusion provided a larger number density of precipitates in the studied alloys. It is well known that during T4 treatment prior to extrusion, most of the insoluble coarse Mg_2_Sn particles in the casting process were dissolved into the matrix; in other words, most of the Sn elements were present in the solid solution states in the matrix. This means that the dynamic precipitation rate of Mg_2_Sn particles was stronger in the extruded alloy with T4 than in the extruded alloy without T4, causing the average size of the DRXed grains to gradually decrease from ~2.96 to ~1.83 μm for both alloys by Zener drag [22,23]. According to the selected area electron diffraction (SAED) pattern (Figure 4a,b), these precipitates with different morphologies could be identified as an Mg_2_Sn phase (face-centered cubic, a = 0.6739 nm). Because the zone axis of the rod-shaped Mg_2_Sn precipitates (00$\bar{1}$) and the Mg matrix (0001) were parallel, it could be confirmed that the rod-shaped Mg_2_Sn precipitate lies on the basal plane, indicating these precipitates are unfavorable to blocking basal <a> dislocation compared to non-basal ones. In contrast, the spherical precipitates in the extruded alloy with T4 showed finer and more uniform distribution, which was relatively effective in basal dislocation drag compared to the rod-like ones [24]. Figure 4c is the schematic illustration of the two different types of precipitates. As is clearly exhibited, the hindering effect of spherical Mg_2_Sn precipitates was stronger than that of the rod-like precipitates, thus leading to a greater strengthening effect. In view of this, T4 should not be omitted in Mg-Sn based alloys with a higher content of alloying elements, in order to achieve superior strengthening effect.

Utilization of the high-resolution TEM (HR-TEM) images obtained from the Mg_2_Sn phases in Figure 4a,b, and the correlative results are shown in Figure 4d,e. It can be concluded that Mg_2_Sn phases have crystalline orientation relationships with the Mg matrix in both alloys, namely: ($\bar{1}$2$\bar{1}$0)_Mg_∥(220)_Mg2Sn_ for the extruded alloy without T4; and (2$\bar{1}$$\bar{1}$0)_Mg_∥(220)_Mg2Sn_ for the extruded alloy with T4. Similar results on the orientation relationship information of fine Mg_2_Sn precipitates with the Mg matrix during extrusion have been reported in extruded Mg-Sn alloy systems [13,25]. 

**Figure 5:**
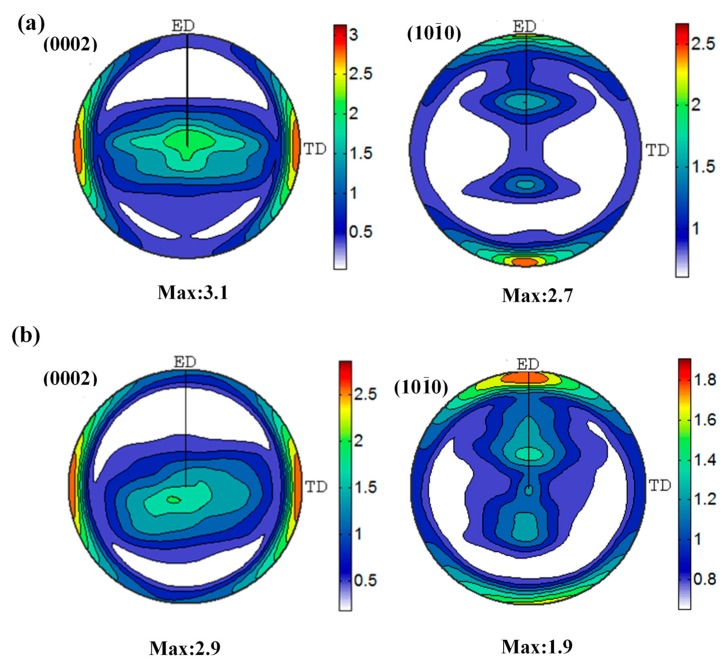
The (0002) and (10$\bar{1}$0) pole figures of extruded TZA822 alloys. (**a**) Without T4 and (**b**) with T4, respectively.

The (0002) and (10$\bar{1}$0) pole figures of the extruded TZA822 alloys are presented in Figure 5. The extruded TZA822 alloys exhibited a texture in which (0002) basal planes and [10$\bar{1}$0] directions were parallel to the ED, which was typical of extruded Mg alloys [26]. The extruded alloy with T4 displayed a weaker texture than did the extruded alloy without T4. It has been reported that the texture of the recrystallized grains was much more randomized than that of the unDRXed grains [23], suggesting that the textural weakening in the extruded alloy with T4 is associated with the decreased fraction of unDRXed grains, which retain strong texture. 

Furthermore, Mg_2_Sn phases in both micro and nano-size precipitated during extrusion provided more randomly oriented nuclei, and contributed to blocking the mobility of dislocations and grain boundaries, thus causing alteration and randomization of the overall texture [21,23]. In other words, the larger the number of dynamic precipitates, the weaker the texture. As exhibited in Figure 4a,b, for example, the extruded alloy with T4 exhibited an abundance of micro and nano-size Mg_2_Sn precipitates, resulting in a weaker texture, which is foreseeable.

### 3.3. Mechanical Properties of the Extruded Alloys

**Figure 6:**
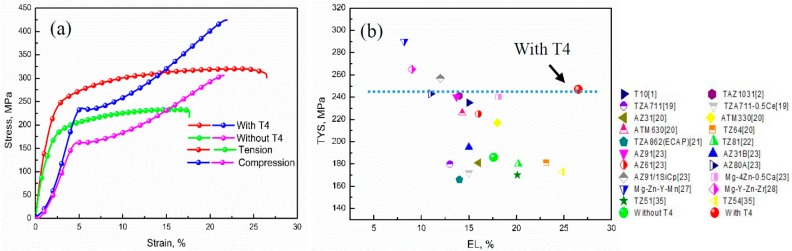
(**a**) Tensile and compressive stress-strain curves of the extruded TZA822 alloys; (**b**) TYS and EL of various Mg based wrought alloys.

Tensile and compressive stress-strain curves of the extruded TZA822 alloys are shown in Figure 6a, and the related properties are also summarized in Table 1. The extruded alloy with T4 exhibited mechanical properties superior to their counterpart without T4. The tensile yield strength (TYS), ultimate tensile strength (UTS), elongation (EL) and compressive yield strength (CYS) for the extruded alloy with T4 were 245 MPa, 320 MPa, 26.5% and 234 MPa, respectively, whereas they were 186 MPa, 231 MPa, 17.6% and 163 MPa for the extruded alloy without T4. The TYS and EL of the studied TZA822 alloys and other wrought Mg alloys are shown in Figure 6b. It could be deduced that the tensile properties of the extruded alloy without T4 were superior to other extruded Mg-Sn based alloys studied in Refs. [19,22,35]. For instance, the yield strength of the extruded alloy without T4 (186 MPa) is 6, 14, 6, 16 and 13 MPa higher than that of Mg-Sn based alloys in Refs 19, 22 and 35 (180, 172, 180, 170 and 173 MPa). This was mainly attributed to the combined effects of refined grain size, as well as finer and more uniform distribution of precipitates. In addition, the extruded alloy with T4 showed a better balance of TYS and EL compared to other available wrought Mg alloys such as Mg-Al-Zn [20,23] and Mg-Zn [23] alloy systems. Interestingly, the extruded alloy with T4 exhibited comparable strength, but larger ductility relative to RE-containing Mg alloys [27,28].

It is noteworthy that the difference in compressive and tensile yield strengths (CYS and TYS) cause tension-compression anisotropy, which can be assessed by the yield asymmetry ratio R (CYS/TYS). The higher R value represented a lower degree of yield asymmetry. In this study, the extruded alloy with T4 had a very high tension to compression ratio of 0.96, which was nearly isotropic in terms of yield, and larger than that of the extruded alloy without T4 (*R* = 0.88). Based on previous reports [3,29], fine grain size and weak basal texture could restrain the activation of {10$\bar{1}$2} twinning under compression to reduce the yield asymmetry. Additionally, the presence of numerous nano-size Mg_2_Sn precipitates were also able to suppress the occurrence of twinning by preventing the motion of dislocation, which could contribute to the decrease of yield symmetry [29,30].

### 3.4. Strengthening Effect 

It is generally believed that the yield strength of wrought magnesium alloys is associated with grain size, dynamic precipitate and texture intensity. Calculations by the Hall-Petch relation for Mg-Sn based alloys [21]: σy=σ0+Kd−12, where *σ_y_* is the YS, *σ*_0_ is the material constant, *K* is the Hall-Petch slope, with a value of 280 MPa μm^−1/2^, indicated that the increment in YS by grain refinement from 2.96 to 1.83 μm was about 44.2 MPa, suggesting a decrease of DRX grain size could partially enhance the strengthening effect. In addition, it is well known that nano-size particles dynamically precipitated during extrusion can serve as obstacles to dislocation movement based on the Orowan mechanism [23,31,32], and that the increase in YS can be calculated by the equation: ΔσP=M0.4Gbπ1−υln(2r−b)λ, where *M* = 1.25 is the Taylor factor, *G* = 16.6 GPa is the Shear modulus, *b* = 0.32 nm is Burgers vector, *ν* = 0.267 is the Poisson ratio, $\bar{r}=\sqrt{\frac{2}{3}}r$, and *r* is the radius of the precipitate. *λ* is the inter-precipitate spacing, which can be calculated by the equation: $\lambda =2\bar{r}(\sqrt{\pi /4f}-1)$, where *f* is the fraction of precipitates. Generally, the strength could be improved by increasing the amount of precipitates, while the increasing size of precipitates would be unfavorable to the strength. By utilizing a measured fraction and size of nano-size Mg_2_Sn precipitates (8.11%, 351.8 nm and 13.87%, 160.7 nm, respectively), the precipitation strengthening *σ*_p_ was estimated to be about 6.1 MPa and 18.3 MPa for the extruded alloys without and with T4, respectively. This indicates that with an increase in the amount of remaining micro-size Mg_2_Sn particles, the strengthening effect of precipitation becomes weaker, thus leading to a deterioration in strength. 

**Figure 7:**
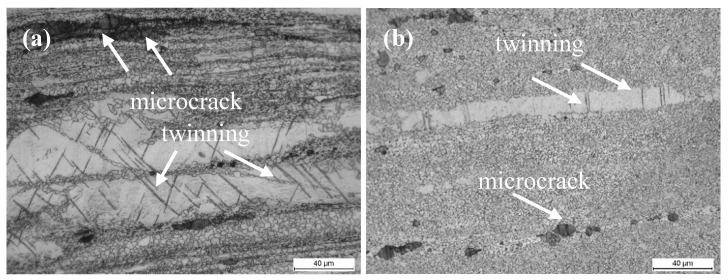
OM from gage sections of fractured tensile samples for the extruded TZA822 alloys in different states: (**a**) without T4 and (**b**) with T4.

It is interesting to note that the extruded alloy with T4 exhibits better ductility than the extruded alloy without T4, although the former exhibits higher strength than the latter. Firstly, the more random texture in the extruded alloy with T4 could lead to more dislocation slip, giving rise to an increment of EL [33]. Secondly, a previous report indicated that double twinning can be easily generated in unDRXed grains for TZA alloy systems during tension, which accelerates cracking due to dislocation pile-ups at the twin-matrix interface [34]. Numerous twins and cracks in the stringers of the coarse Mg_2_Sn particles could be observed in the fractured sample without T4 (Figure 7a), which could provide initiation sites for microcracks. Finally, the strain hardening exponents *n* ($\sigma =K\varepsilon^{n}$, where *K* is the strength coefficient) were calculated as 0.18 and 0.21 for the extruded alloys without and with T4, respectively. Generally, higher *n* values led to lower sensitivity to strain localization, and hence a greater EL [35].

## 4. Conclusions

Varying Mg_2_Sn contents containing TZA822 alloys prior to extrusion were subjected to different pre-treatments (without and with T4), and the strengthening response due to micro and nano-size Mg_2_Sn precipitates in the extruded TZA822 alloys were investigated. Conclusions were drawn as follows:

(1) The morphology of nano-size Mg_2_Sn precipitates exhibited a significant change in basal plane from rod-like to spherical due to the decrement in the fraction of micro-size particles before extrusion.
(2) The spherical Mg_2_Sn precipitates provided a much stronger strengthening effect than the rod-like precipitates, which was ascribed to uniform dispersion and refinement of spherical precipitates to effectively hinder basal <a> dislocation slip.
(3) The refined precipitate microstructure led to high TYS of 245 MPa, UTS of 320 MPa and EL of 26.5% while keeping a lower degree of yield asymmetry (R = 0.96) in the extruded TZA822 alloy with T4. The balance of strength and ductility for the studied alloys was comparable to that of RE-containing Mg alloys.

## Figures and Tables

**Table 1:** Microstructural characteristics and mechanical properties of extruded TZA822 alloys.

State	Microstructure			Mechanical Properties					
	*F_DRX_* (%)	*d_DRX_* (μm)	Texture Intensity	TYS (MPa)	UTS (MPa)	EL (%)	CYS (MPa)	YR	n
Without T4	86.2 ± 3.1	2.96 ± 0.34	3.1	186 ± 3	231 ± 4	17.6 ± 0.3	163 ± 3	0.88	0.18
With T4	96.1 ± 1.8	1.83 ± 0.26	2.9	245 ± 2	320 ± 2	26.5 ± 0.2	234 ± 3	0.96	0.21

*F_DRX_* and *d_DRX_* represent the volume fraction and average grain size of DRX grains, respectively. Texture Intensity represent intensity of (0002) basal planes. TYS, UTS, EL, CYS represent tensile yield strength, ultimate tensile strength, elongation and compressive yield strength. YR (yield asymmetric ratio) = CYS/TYS; n represent work hardening exponents.
